# Extraskeletal Myxoid Chondrosarcoma with Small Bowel Metastasis Causing Bowel Obstruction

**DOI:** 10.1155/2012/621025

**Published:** 2012-11-18

**Authors:** Ernesto Bustinza-Linares, Francisco Socola, Vinicius Ernani, Shelly A. Miller, Jonathan C. Trent

**Affiliations:** ^1^Division of Hematology and Oncology, Department of Medicine, Sylvester Comprehensive Cancer Center, University of Miami, Miami, FL 33136, USA; ^2^Division of Internal Medicine, Department of Medicine, Miller School of Medicine, University of Miami, Miami, FL 33136, USA

## Abstract

A 28-year-old female with history of chest wall extraskeletal myxoid chondrosarcoma (EMC) presented to the emergency department complaining of two weeks of lightheadedness and fatigue. Laboratories showed hemoglobin of 7.6 g/dL and a positive hemoccult test. Upper and lower endoscopies were unremarkable, and the patient was discharged after blood transfusion. The next day she returned to the ED with left-sided weakness and perioral numbness. Brain CT scan revealed a 6 cm right frontal mass with midline shift and edema that required urgent craniotomy with resection of a hemorrhagic tumor. The patient continued dropping her hemoglobin, and CT scans showed a rounded 3 cm small bowel mass in the mid ileum. Repeat upper endoscopy revealed a 2 × 2 cm ulcerated mass in the fourth portion of the duodenum. The patient was taken to the operating room and was found to have two lesions; one in the distal duodenum and a second one in the mid ileum causing small bowel intussusception. Pathology was consistent with metastatic EMC grade 2/3, involving the bowel and mesenteric fat. Extraskeletal myxoid chondrosarcoma (EMC) is a rare soft-tissue sarcoma with unique features that distinguishes, it from other sarcomas. It has been often described as a low-grade sarcoma although there are certain characteristics like high mitotic activity and the presence of focal regions of Ki67 staining above 25% that correlate with aggressive behavior of the tumor. This is the first case of EMC metastatic to the small bowel to be reported to the medical community.

## 1. Introduction

**Figure 1:**
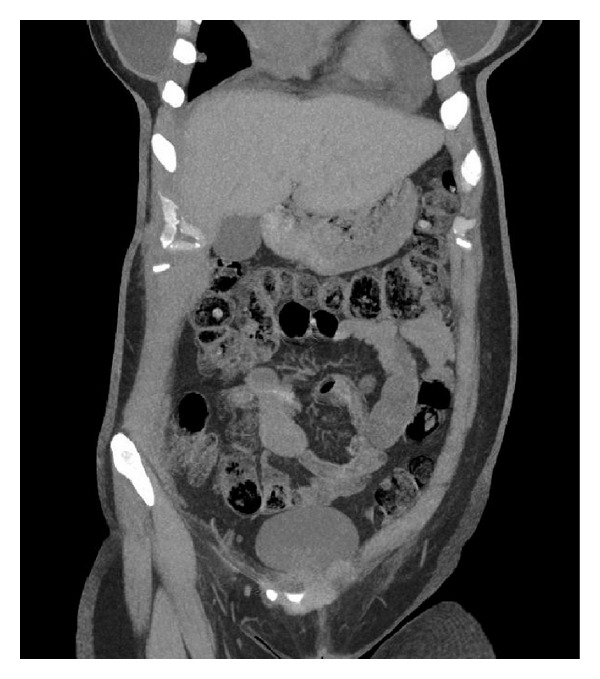
Abdominal CT scan with evidence of small bowel intussusception.

**Figure 2:**
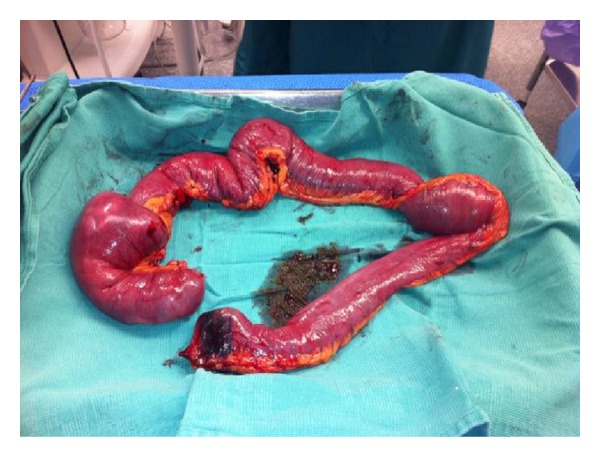
Surgical specimen of small bowel with metastatic extraskeletal myxoid chondrosarcoma, grade 2/3, to the bowel and mesenteric fat with secondary intussusception and bowel obstruction.

**Figure 3:**
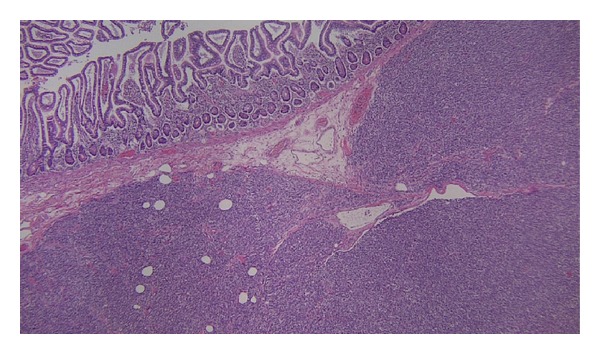
The largest metastatic deposits involve the full thickness of the bowel wall and demonstrate luminal ulceration and have caused intussusception and obstruction. The overwhelming majority of the tumor is viable. Arterial and venous invasion are prominent.

**Figure 4:**
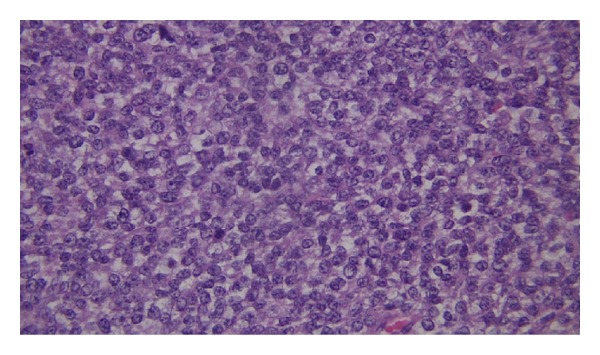
The tumor is hypercellular and mitotically active.

A 28-year-old female with history of chest wall extraskeletal myxoid chondrosarcoma (EMC) presented to an outside hospital with two weeks of lightheadedness, and fatigue. Initial laboratories showed hemoglobin of 7.6 g/dL and a positive guaiac test. The patient underwent upper and lower endoscopies that were found to be unremarkable. Patient was discharged home after blood transfusion and scheduled for outpatient follow-up visit. The next day the patient returned to the emergency room with sudden left facial droop, left upper extremity weakness and perioral numbness. Emergent brain CT scan revealed a 6 cm right frontal mass with midline shift and edema. The patient was stabilized and transferred to our institution. She was taken to the operating room for an urgent right frontotemporoparietal craniotomy with resection of hemorrhagic tumor. During her postoperative period the patient continuously dropped her hemoglobin and a fecal occult blood test persisted being positive. For that reason, CT scans revealed a rounded 3 cm small bowel lesion in the mid ileum (Figure 1). Repeated upper endoscopy showed an ulcerated mass measuring 2 × 2 cm in the fourth portion of the duodenum. Due to persistent anemia and gastrointestinal bleeding, the patient case was presented to the multidisciplinary tumor board, and it was recommended to proceed with palliative small bowel tumor resection. Meanwhile, the patient successfully completed adjuvant radiation therapy to the frontal metastasis resection bed. Four days before the scheduled surgery, the patient developed nausea, vomiting and abdominal distention. The patient was taken to the operating room and found to have two separate lesions involving the small bowel. The first one was in the fourth portion of the duodenum and the second one in the mid ileum causing small bowel intussusception (Figure 2). Sarcomatosis throughout the peritoneum was also found. Pathology from the metastatic brain lesion and the small bowel lesions were reviewed by a sarcoma pathologist and found to be consistent with metastatic extraskeletal myxoid chondrosarcoma grade 2/3, to the bowel and mesenteric fat with secondary intussusception and bowel obstruction. The tumor was hypercellular and high grade (Figures 3 and 4). The largest metastatic deposits involved the full thickness of the bowel wall and demonstrated luminal ulceration resulting in intussusception and obstruction. The mesenteric fat contained separate, small metastatic deposits. Arterial and venous invasion were prominent, and the bowel resection margins were negative. Immunohistochemistry assays performed at our institution revealed immunohistochemical presence of CD99, FLI1, INI1, Synaptophysin (weak) and EMA (rare focal positive cells). The tumor was negative for Keratin, GFAP, CD45, CD31, S100, CD34, Tdt, neuron specific enolase, and desmin. Our cytogenetics and molecular diagnostic laboratory examined the tissue by Interphase FISH with LSI EWSR1 break apart probe (Abbott, IL, USA) (22q12) with 200 cells scored finding nuc ish(5′EWSR1, 3′EWSR1)x2(5′EWSR1 con 3′EWSR1x2) consistent with no EWSR1 gene rearrangement. The mitotic rate was 23 per 10 high power fields in the tumor resected from the brain.

## 2. Discussion 

Extraskeletal myxoid chondrosarcoma (EMC) is a rare soft-tissue sarcoma with unique clinicopathological, immunohistochemical, and genetic features that distinguishes it from other sarcomas [1–3]. EMC has been estimated to account only for 2.5% of all soft-tissue sarcomas [4]. EMC has a male to female ratio of 2 : 1 and a median age at presentation of 52 (6–89) with tumor sizes ranging from 1 to 25 cm with a median of 7 cm [1]. It usually arises in the deep subcutis or deeper soft-tissues, with 80% of the tumors presenting in the proximal extremities or limb girdles and only 20% in the trunk [1]. Less usual sites of presentation have been described in the literature including knee joint [5], vulvae [6], and labium majus [7]. Unusual clinical presentations have been reported including sensorimotor polyneuropathy associated with anti-Hu antibodies [8]. The most common metastatic sites described are lung, bones, lymph nodes, and soft tissues [3]. Other less usual sites of metastasis are intracranial [9] and pancreatic [10]. The local recurrence rate ranges between 37% and 48% and distant metastasis ranges between 26% and 46% depending on the published series [1, 2]. The EMC estimated 5-, 10-, and 15-year survival rates reported are 90%, 70%, and 60%, respectively. Historically these tumors are often described as a low-grade sarcoma although their clinical behavior is unpredictable [1]. Moreover, it has been very well documented that certain characteristics can determine an EMC to behave as a high-grade tumor. Characteristics that correlate with aggressive behavior include: tumor size more than 10 cm, atypical features such as anaplasia and rhabdoid phenotype, as well as, presence of focal regions of Ki-67 staining above 25% (Ki67 “hot spot”) which strongly correlate with decreased metastasis-free and overall survival [3, 11, 12]. High cellularity correlates with poor overall survival and mitotic activity; more than 2 per 10 hpf or overall Ki67 more than 10% correlates with decreased metastasis-free survival [3]. Patients with high-grade EMC have a shorter median survival than those patients with low- or intermediate-grade disease [11, 13]. Cytogenetic studies demonstrated the presence of a recurrent translocation t(9;22)(q22;q12) that results in the fusion of the EWSR1 gene on chromosome 22 with NR4A3 (TEC, CHN, NOR1) gene on chromosome 9. This gene fusion encodes a fusion protein in which the C-terminal RNA-binding domain EWSR1 (EWS) is replaced by the entire NR4A3 (TEC) protein. This fusion protein is a potent transcriptional activator [14]. Other clonal chromosome abnormalities have been reported including del(22)(q12-13) and variant translocations, including t(9;17)(q22;q11-12), t(7;9;17)(q32;q22;q11), and t(9;15)(q22;q21) [15]. There is no evidence of efficacy of standard chemotherapy in EMC [16]. In the largest series reported, twenty-one patients were treated with chemotherapy with a combined total of 39 courses of chemotherapy. Twenty-five different regimens were administered with doxorubicin-containing regimens comprising the largest group. No radiologic responses were noted (CR or PR). The best objective response was stable disease for more than 6 months in 2 patients [2]. Recently, a randomized, double-blind, placebo-controlled phase III study of 372 patients with metastatic soft-tissue sarcomas using Pazopanib (Votrient, GlaxoSmithKline, Brentford, Middlesex, TW8 9GS, United Kingdom) reported a median progression-free survival of 4.6 months in the therapy group compared with 1.6 months in the placebo group (*P* < 0.0001). The overall survival was 12.5 months with Pazopanib versus 10.7 months with placebo but it was not statistically significant (p < 0.25) [17].

To the best of our knowledge, this is the first report of EMC metastatic to the small bowel. Our case has several peculiar characteristics including the lack t(9;22)(q22;Q12) mutation, the very high mitotic rate, and the significant percentage of viable tumor at the time of resection which translated into a particularly aggressive behavior of the disease. EMC is a rare sarcoma that should be evaluated by a multidisciplinary team including an experienced pathologist as soon as possible to avoid delays in the surgical removal of the tumor which at this point is the only effective therapeutic option available. Patients with metastatic disease should be evaluated for participation in clinical trials.
